# Food-Associated *Lactobacillus plantarum* and Yeasts Inhibit the Genotoxic Effect of 4-Nitroquinoline-1-Oxide

**DOI:** 10.3389/fmicb.2017.02349

**Published:** 2017-11-28

**Authors:** Roberta Prete, Rosanna Tofalo, Ermanno Federici, Aurora Ciarrocchi, Giovanni Cenci, Aldo Corsetti

**Affiliations:** ^1^Faculty of Bioscience and Technology for Food, Agriculture and Environment, University of Teramo, Teramo, Italy; ^2^Laboratory of Microbiology, Department of Chemistry, Biology and Biotechnology, University of Perugia, Perugia, Italy

**Keywords:** *Lactobacillus plantarum*, yeasts, acid-bile tolerance, 4-nitroquinoline-1-oxide, antigenotoxicity, SOS-chromotest

## Abstract

Lactic acid bacteria and yeasts, representing the prevailing microbiota associated with different foods generally consumed without any cooking, were identified and characterized *in vitro* for some functional properties, such as acid-bile tolerance and antigenotoxic activity. In particular, 22 *Lactobacillus plantarum* strains and 14 yeasts were studied. The gastro-intestinal tract tolerance of all the strains was determined by exposing washed cell suspensions at 37°C to a simulated gastric juice (pH 2.0), containing pepsin (0.3% w/v) and to a simulated small intestinal juice (pH 8.0), containing pancreatin (1 mg mL^-1^) and bile extract (0.5%), thus monitoring changes in total viable count. In general, following a strain-dependent behavior, all the tested strains persisted alive after combined acid-bile challenge. Moreover, many strains showed high *in vitro* inhibitory activity against a model genotoxin, 4-nitroquinoline-1-oxide (4-NQO), as determined by the short-term method, SOS-Chromotest. Interestingly, the supernatants from bacteria- or yeasts-genotoxin co-incubations exhibited a suppression on SOS-induction produced by 4-NQO on the tester strain *Escherichia coli* PQ37 (*sfi*A::*lac*Z) exceeding, in general, the value of 75%. The results highlight that food associated microorganisms may reach the gut in viable form and prevent genotoxin DNA damage *in situ.* Our experiments can contribute to elucidate the functional role of food-associated microorganisms general recognized as safe ingested with foods as a part of the diet.

## Introduction

Over the past decades, the food industry has been revolutionized toward the production of functional foods due to an increasing awareness of the consumers on the positive role of food in well-being and health. In general, the term refers to a food that has been modified in some way to become “functional” (Noomhorm et al., 2014). One way in which foods can be modified is by addition of probiotics, but also fermented foods containing “live or active bacteria” or yeasts that provide benefits to gut health, might also qualify as health-promoting foods (Shenderov, 2013; Hill et al., 2014). In this perspective, the ability of food-borne microbes to exert antigenotoxic properties and to make a protective role at gastro-intestinal level by inhibiting the biological activity of genotoxic compounds is considered a functional property (Raman et al., 2013). Recently, several studies are focusing on food-associated microorganisms that, as a normal component of the diet, can interact with the human host and can be related to a reduced colon cancer incidence from environmental factors, such as dietary and endogenous xenobiotics (Jeffery and O’Toole, 2013; Raman et al., 2013; David et al., 2014). Currently, the antigenotoxic and antimutagenic activities are considered among the functional properties for characterizing probiotic microorganisms (Cenci et al., 2008; Trotta et al., 2012) and lactic acid bacteria (LAB) isolated from fermented foods have been found to decrease the genotoxicity of some chemical compounds (Burns and Rowland, 2000; Caldini et al., 2008; Novak et al., 2015). An interesting aspect to be considered is the possible contribution of traditional fermented foods and their microbial components to the physiology of the gastro-intestinal tract (GIT) and its protection from endogenous and exogenous risk factors. In fact, fermented foods are considered rich sources of probiotics or microorganisms with functional properties as antimicrobial and antioxidant properties, fibrinolytic activity, poly-glutamic acid, degradation of antinutritive compounds peptide and vitamins production which may be important criteria for selection of starter culture(s) to be used in fermented functional foods (Capozzi et al., 2012; Walia et al., 2014; Tamang et al., 2016). Actually, by considering the vast food-borne microbiota, it is well-documented that lactobacilli and bifidobacteria may have a role in prevention and protection from genotoxic compounds using *in vitro* and *in vivo* models (Cenci et al., 2002; Burns and Rowland, 2004; Caldini et al., 2005; Dominici et al., 2014; Novak et al., 2015). The most widely studied strains belong to the species *Lactobacillus casei, Lactobacillus acidophilus, Lactobacillus rhamnosus*, and *Lactobacillus delbrueckii* and other genera, such as *Bifidobacterium* and *Bacillus* (Caldini et al., 2002; Cenci et al., 2002; Raipulis et al., 2005), but the ability of *Lactobacillus plantarum* species to counteract genotoxic compounds in the gut is still not well-investigated (Corsetti et al., 2015).

Moreover, only few information are available about the possible antigenotoxicity of yeast strains, even though they are commonly present in many traditional fermented foods as well as in the gastrointestinal tract as commensals. However, yeasts are considered as microorganisms that may improve human health (Moslehi-Jenabian et al., 2010). Psani and Kotzekidou (2006); Silva et al. (2011), and Botta et al. (2014), evaluated the probiotic characteristics of table olive related yeasts. At present, the functional properties of food-borne yeasts have been focused especially for strains belonging to the genus *Saccharomyces* (Fleet, 2007), such as *S. cerevisiae* and *S. boulardii* (Czerucka et al., 2007; Sourabh et al., 2011). Probiotic properties have been reported also for the yeast species *Kluyveromyces marxianus*, frequently isolated from dairy products (Lisotti et al., 2013; Tofalo et al., 2014; Qvirist et al., 2016).

The question arises whether other yeast species possess functional properties as well, considering that some strains isolated from several sources (including tropical fruit and plants) exhibited lipolytic and proteolytic activities, tolerated low pH and survived to simulated gastric and intestinal fluids, so could be considered probiotics candidates (Tamang et al., 2016). Thus, the interest in using yeasts as a novel nutrient fortification is relevant (Fleet, 2007; Kogan et al., 2008).

Based on the above premises, the aim of this work was to evaluate the antigenotoxic activity of 22 strains of *L. plantarum* and 15 strains of 10 yeasts species, isolated from various foods against 4-NQO, a nitroaromatic genotoxin which produces strand scission and formation of charge-transfer adducts on DNA (Fann et al., 1999; Nair et al., 2000).

Moreover, in order to hypothesize if those microorganisms should act against genotoxic compounds in the gut, their capability to survive against the harsh conditions of the GIT, was also evaluated by an *in vitro* acid-bile tolerance test.

## Materials and Methods

### Origin of Bacteria and Yeasts Strains

All the microorganisms belong to the Microbial Collection of the Bioscience Faculty (Teramo University, Italy). *L. plantarum* WCSF1 and ATCC^®^14917^TM^ and two strains with documented probiotic activities, *L. plantarum* IMC 510^®^ and IMC 513^®^ (Synbiotec, Camerino, Italy), were used as reference strains for lactobacilli and a probiotic *S. boulardii* (Codex^®^, Zambon-Italia) as a reference strains for yeasts.

All the food-borne microorganisms were isolated from different foods, including various samples with different features (brands, food origin, time of ripening, etc.). In particular, LAB were isolated from three different fermented foods (table olives, sourdoughs, and raw-milk cheeses) and yeasts were isolated from different foods, mainly fruits and vegetables (**Table 1**). The isolation procedures were performed as previously described (Schirone et al., 2013). LAB and yeasts isolates were stored at -80°C in the Culture Collection of the Faculty of Bioscience and Technology for Food, Agriculture and Environment (University of Teramo).

**Table 1:** Lactic acid bacteria (LAB) and yeasts used in this study.

Species	*N*	Strains	Source
LAB			
*Lactobacillus plantarum*	22	05, 013, N14, C9O4, C9S2 21B, CF1 LAB1, LAB30, LAB32, LAB40, LAB49, LAB62 LT21, LT52, LT53, LT99, LT100 ATCC^®^14917^TM^ WCSF1 IMC 510^®^, IMC 513^®^	Table olives Sourdoughs Raw-milk cheeses Raw-milk cheeses Pickled cabbage Human saliva Synbiotec s.r.l.
Yeasts			
*Debaryomyces hansenii*	2	LG2, LG15	Raw-milk cheeses
*Wickerhamomyces anomalus*	2	LUL14 TO8	Lupins Topinambur
*Pichia fermentans*	2	TO1, TO10	Topinambur
*Torulaspora delbrueckii*	2	TO2, TO3	Topinambur
*Hanseniaspora uvarum*	1	TO5	Topinambur
*Metschnikowia* aff. *fructicola*	2	RIB1, RIB3	Currant
*Metschnikowia raukaufii*	1	LAM3	Salmon
*Candida apicola*	1	UV10	Grapes
*Meyerozyma guilliermondii*	1	PR1	Plum
*Saccharomyces boulardii*	1	Codex^®^	Codex^®^ (Zambon Italia)

### Identification and Strain Typing

Genomic DNA was extracted from LAB and yeasts cultures as reported by De Los Reyes-Gàlivan et al. (1992) and Querol et al. (1992), respectively. The LAB isolates were identified by sequencing the 16S rRNA gene according to Corsetti et al. (2008), proved by *rec*A gene multiplex PCR assay. Yeasts were identified by the D1/D2 domain of the 26S rRNA gene according to Kurtzman and Robnett (1998). LAB and yeasts were differentiated at strain level by RAPD (Randomly Amplified Polymorphic DNA)-PCR performed as previously described (Andrighetto et al., 2000; Tofalo et al., 2009) using the primer M13 (5′-GAGGGTGGCGGTTCT-3′).

### Chemicals

The direct-acting genotoxin 4-NQO (CAS no. 56–57–5) was obtained from Sigma-Aldrich (St. Louis, MO, United States). A stock solution (1 mg/ml) was prepared, for 4-NQO in dimethyl sulfoxide (DMSO) and diluted in water before the tests. The substrates *o*-nitrophenyl-β-D-galactopyranoside (ONPG) and *p*-nitrophenylphosphate (PNPP), for colorimetric evaluation of β-galactosidase and alkaline phosphatase respectively, were purchased from Sigma Aldrich.

### Simulated Gastro-Intestinal Conditions

Simulated gastric fluid (SGF) and simulated intestinal fluid (SIF) were prepared fresh daily in order to evaluate the acid-bile tolerance of all strains. Overnight cultures of LAB and yeasts on MRS and YPD liquid medium respectively, were harvested by centrifugation (6000 × *g* for 15 min). The pellets were re-suspended in SGF (pH 2.0) containing pepsin (1 mg/ml), and maintained at 37°C for 2 h. After acid pre-incubation cells were centrifuged again and re-suspended in SIF (pH 7.4) containing pancreatin (1 mg/ml) and bovine bile (0.5% w/v) dissolved in saline, and maintained at 37°C for 3 h (Haller et al., 2001; Cenci et al., 2006). Pepsin and pancreatin were obtained from Sigma-Aldrich; bile extract, containing conjugated glycholate and taurocholate sodium salts, from Oxoid (LP0055, Oxoid, Ltd., Basingstoke, United Kingdom). After acid-bile challenge samples were removed, serially diluted, and plated on MRS and YPD containing 2% agar (Oxoid, Ltd., Basingstoke, United Kingdom) to determine cell viability. All the strains was tested in triplicate and the survival rate of all the tested microorganisms was estimated by evaluating the ratio between viable cells after the acid or bile challenge and viable cells of the untreated controls. The acid tolerance and acid-bile tolerance percentage was calculated from the *L. plantarum* and yeasts survival rate.

### Genotoxin-Cells Incubation

The cell-genotoxin interaction was evaluated using the following co-incubation protocol. LAB and yeasts were cultured, in triplicate, in MRS and YPD broth (Oxoid) at 37 and 30°C for 24 h, respectively. Bacterial and yeasts cultures were washed (6000 × *g* for 15 min) and resuspended in physiological saline until 10^8^–10^9^ cell/ml. The 4-NQO was added in a final concentration of 0.1 mM and co-incubation was maintained at 37°C for 150 min (under shaking) according to Caldini et al. (2002). After co-incubation the residual genotoxic activity was determined on filtered (0.45 μm Sartorius membrane) supernatants and analyzed for residual genotoxic and mutagenic activity as for UV-visible spectrophotometric profiles to evaluate genotoxin modification.

### Genotoxicity Evaluation by the SOS-Chromotest

4-NQO genotoxicity and its residual activity after cell co-incubation were carried out using SOS-Chromotest (Quillardet and Hofnung, 1985, 1993). This method is a useful, rapid screening for genotoxicity and has several advantages including sample preparation, short analysis time and agreement with other biological assays for mutagenicity and genotoxicity (Rosenkranz et al., 1999; Cenci et al., 2005). The SOS-Chromotest was performed using *Escherichia coli* PQ37 (*sfi*A::*lac*Z, *uvr*A, rfa, Pho^c^) as tester strain, which carries a *sfi*A::*lac*Z gene fusion and has deletion of the normal lac region. *E. coli* PQ37, supplied by the Institute Pasteur (Paris, France), was grown in L-broth (10 g/l tryptone, 5 g/l yeast extract, and 10 g/l NaCl) and LA-broth (supplemented with 20 μg/ml ampicillin). Genotoxicity in samples was detected measuring the activation of SOS-response of tester organism by evaluating β-galactosidase (BG) induction and alkaline phosphatase (AP) constitutive expression. The constitutive AP production of *E. coli* PQ37 is an indicator of protein synthesis in the presence of sub-lethal doses of genotoxin. The SOS induction factor (IF_sos_) is defined as the BG/AP ratio of the sample under analysis, divided by the same ratio of negative control. Positive and negative controls were prepared in saline solution, with or without genotoxin, respectively. The enzyme activities were detected colorimetrically according to details given in a previous study (Caldini et al., 2002). The genotoxicity inhibition (GI) produced by different strains was also given in percentages considering the IFsos of positive controls (genotoxin without lactobacilli co-incubation) as 100%. The threshold for strain antigenotoxicity was fixed as follows: GI 75% high active, GI between 25 and 75% moderately active, GI 25% inactive (Corsetti et al., 2008). The experiments were carried out in the absence of exogenous metabolic activation of genotoxins.

### Physico-Chemical Analysis

Supernatants of genotoxin-treated cultures were examined by absorbance profiles in the range 230–400 nm obtained by a microplate reader (Infinite 200 Pro, Tecan icontrol), in order to prove chemical-biological interactions.

## Results

### Identification and Strain Typing

Twenty-two strains were identified as *L. plantarum* by sequencing the 16S RNA gene and by *rec*A multiplex PCR assay (data not shown), having the PCR amplicons of 318 bp (Torriani et al., 2001). Regarding yeast isolates, 14 food-associated yeast strains were selected for this study. From the D1/D2 domain of 26S rRNA analysis, they belong to nine different species: *Debaryomyces hansenii* (2), *Wickerhamomyces anomalus* (2), *Metschnikowia* aff. *fructicola* (2), *Pichia fermentans* (2), *Torulaspora delbrueckii* (1), *Candida apicola* (1), *Meyerozyma guilliermondii* (1), *Hanseniaspora uvarum* (1), and *Metschnikowia raukaufii* (1). All the isolates were differentiated at strain level by RAPD-PCR assay (data not shown). Strains used in the study and their relative species are reported in **Table 1**.

### Tolerance to Gastro-Intestinal Transit

**FIGURE 1:**
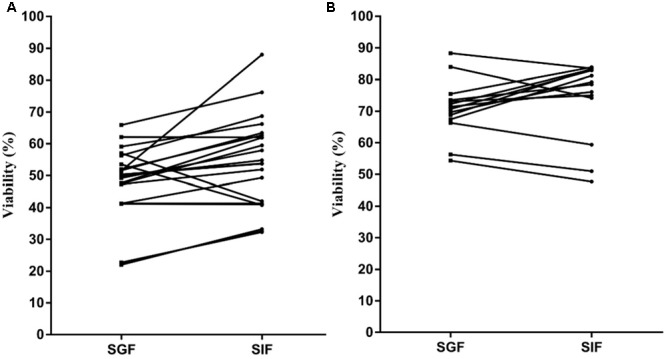
Viability of *Lactobacillus plantarum* **(A)** and yeast **(B)** strains after consecutive exposure to simulated gastric fluid (SGF) and simulated intestinal fluid (SIF).

**FIGURE 2:**
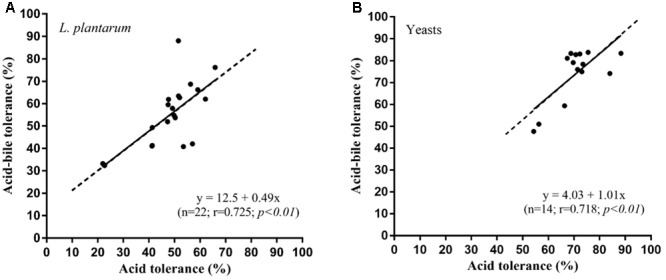
Relationship between acid-tolerance and combined acid-bile tolerance in *L. plantarum* **(A)** and yeast **(B)** strains.

*Lactobacillus plantarum* and yeasts tolerance toward gastro-intestinal transit was as follows. The viability of the *L. plantarum* strains showed an appreciable reduction (**Figure 1A**), ranging from 20 to 65% after exposure to SGF. For most strains, a clear recovery was observed after incubation in SIF, reaching 30 to 90%. The yeasts viability (**Figure 1B**) in SGF resulted higher (55–90%) compared with that of lactobacilli and the same level was almost maintained in SIF. The results obtained showed a significant correlation (*p* < 0.01) between acid tolerance and combined acid bile tolerance in both *L. plantarum* (**Figure 2A**) and yeast (**Figure 2B**) strains.

### Antigenotoxic Properties

**FIGURE 3:**
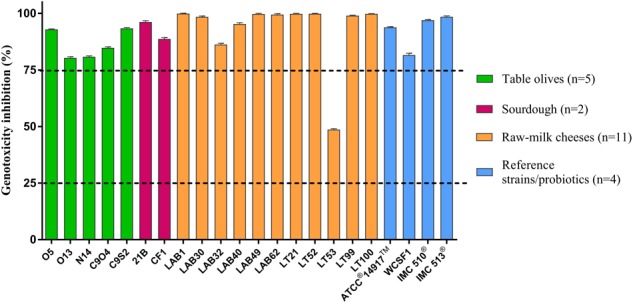
*In vitro* antigenotoxicity of *L. plantarum* strains toward 4-NQO in the SOS-Chromotest. Percent genotoxicity inhibition was calculated from residual activity (SOS induction factor) evaluated on supernatants in relation to that of a positive control. The dashed lines show the threshold levels for antigenotoxicity. Data are shown as means from three replicates. Bars indicate the standard deviation (SD).

**FIGURE 4:**
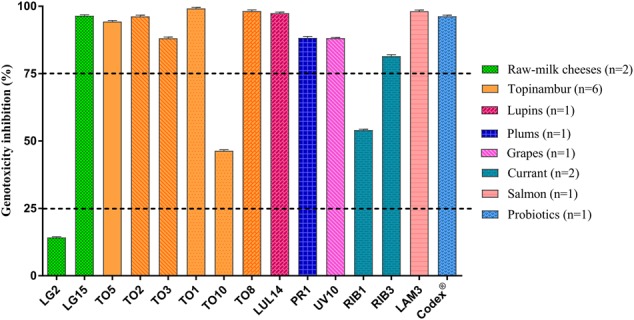
*In vitro* antigenotoxicity of yeasts species and strains toward 4-NQO in the SOS-Chromotest. Percent genotoxicity inhibition was calculated from residual activity (SOS induction factor) evaluated on supernatants in relation to that of a positive control. The dashed lines show the threshold levels for antigenotoxicity. Data are shown as means from three replicates. Bars indicate the standard deviation (SD). *Saccharomyces boulardii* Codex^®^ was used as reference strain and different column patterns show different species (LG2, LG15 *Debaryomyces hansenii*; LUL14, TO8 *Wickerhamomyces anomalus*; UV10 *Candida apicola*; TO1, TO10 *Pichia fermentans*; RIB1, RIB3 *Metschnikowia* aff. *fructicola*; TO2, TO3 *Torulaspora delbrueckii*; PR1 *Meyerozyma guilliermondii*; TO5 *Hanseniaspora uvarum*; LAM3 *M. raukafii*).

The genotoxic effect of 4-NQO in the SOS-Chromotest was strongly reduced under *in vitro* co-incubation with *L. plantarum* and yeasts. **Figure 3** shows that all the *L. plantarum* strains, with one exception (LT53), were effective in reducing 4-NQO activity, exhibiting more of 75% genotoxicity inhibition. A similar behavior was observed for yeasts: only three strains (LG2, TO10, and RIB3) that had lower effect against genotoxin as shown in **Figure 4**.

Moreover, the results obtained with reference probiotic strains (*L. plantarum* IMC 510^®^, *L. plantarum* IMC 513^®^ and *S. boulardii* Codex^®^) were similar to those obtained with the majority of food-borne lactobacilli and yeasts.

### Genotoxin Modification

Evident modifications of spectroscopic properties of 4-NQO were observed in the co-incubation supernatants after the exposure of the genotoxin to live cells of *L. plantarum* and yeasts with antigenotoxic activity (**Table 2**). In particular, the spectroscopic analysis revealed that the typical maximum absorbance peak of 4-NQO (365 nm) is moved to a lower wavelength (hypsochromic shift). The maximum absorbance was on average decreased of 17.3 ± 2.1 nm (range: 15–21) for *L. plantarum* strains. Similarly, for strains of the different yeast species the average absorbance decrease was 13.8 ± 3.2 nm (range: 7–19) showing shifts lower than those of lactobacilli. The different wavelength of peaks recorded after 4-NQO-*L. plantarum* and yeasts co-incubation supports species and strain dependent behaviors, suggesting the possible involvement of different genotoxicity inhibition mechanisms. In the case of food-borne lactobacilli, the hypsochromic shifts and the inhibition of 4-NQO activity were analogous to those found in probiotic and collection strains used as a reference. Relative to yeasts, although the absorbance shift produced by the probiotic *S. boulardii* was much lower than that of other strains, the levels of genotoxicity inhibition were equivalent to those of other yeasts.

**Table 2:** Relationship between 4-NQO spectroscopic modifications and genotoxicity inhibition after its co-incubation with *L. plantarum* and yeast strains.

Strains	4NQO spectroscopy after strain co-incubation	
	λ_max_ (nm)	Δ λ_max_^a^ (nm)	Genotoxicity inhibition (%)^b^
**Lactobacilli (*n* = 22)**			
LAB1 ATCC^®^14917^TM^, WCSF1, IMC 510^®^, LAB49, LT21	352 350	13 15	99.9 94.7
C9S2, CF1, LAB40, LAB62, LT52, LT53, LT99, LT100	348	17	78.3
IMC 513^®^, 013, N14, C904, LAB30, LAB32	346	19	87.9
O5, 21B	344	21	92.9
**Yeasts (*n* = 15)**			
Codex^®^	358	7	93.6
LG2, TO8	354	11	75.2
LUL14, TO1, TO10, TO5, RIB1, LAM3, PR1	352	13	73.3
UV10	350	15	88.1
LG15, TO2	348	17	96.3
TO3, RIB3	346	19	84.7

*^a^4NQO λ_*max*_ without cell co-incubation corresponds to 365 nm.*

*^b^Mean value.*

## Discussion

Some fermented foods and beverages are well-known to possess health benefits due to presence of functional microorganisms (Ross et al., 2002; Tamang et al., 2016). LAB mostly *Enterococcus, Lactobacillus, Lactococcus, Leuconostoc, Pediococcus* and *Weissella* species, are widely used in food fermentation production, including dairy-products, fermented meats, and vegetables (Holzapfel and Wood, 2014). In addition, many yeasts species from fermented foods, alcoholic beverages and non-food mixed amylolytic starters have been characterized in both their technological and beneficial role (Tamang and Fleet, 2009). Bacteria and yeasts in fermentation processes could also enhance the bioavailability of nutrients, the organoleptic and sensorial quality of food other than impart the preservation with the production of antimicrobial and antioxidant compounds (Tamang et al., 2016). Moreover, some of these microorganisms from traditional fermented foods could improve the food safety and quality by degrading toxic components such as genotoxic and mutagenic substances (Farhad et al., 2010; Bourdichon et al., 2012). Therefore, it is well-known that fermented foods, especially dairy products (i.e., fermented milks and yogurt) may constitute a good reservoir of microorganisms for the production of functional foods with probiotic activity, but nowadays the necessity for novel and non-dairy probiotics products is increasing, as reported for table olives (Argyri et al., 2013; Botta et al., 2014).

In this work, the ability of 36 microorganisms, 22 lactobacilli belonging to the *L. plantarum* species and 14 yeasts from different species mainly isolated from table olives, cheeses, sourdoughs, and fruits were studied for their antigenotoxic effects. We confirmed that all the strains possessed high inhibitory activity against 4-NQO, as previously reported for LAB species isolated from milk-based fermented foods (yogurt and fermented milk) (Caldini et al., 2005; Trotta et al., 2012).

In particular, *L. plantarum* is considered a flexible and versatile species and has been found as dominant in several environmental niches and it is frequently encountered as a natural inhabitant of the human GIT (Corsetti et al., 2015). Despite the importance of this species in fermented foods as a starter culture, recently the potential use of *L. plantarum* as a probiotic is also increasingly gaining attention. In this respect, *L. plantarum* species could be preferred above other LAB, for high performance in the GIT, high-level genetic accessibility and low-cost production (Ahrne et al., 1998; Holzapfel et al., 1998; Cunningham-Rundles et al., 2000; de Vries et al., 2006).

Focusing on yeasts, this work gives the opportunity of carrying out a research on functional properties of yeasts, whereas recently the question arises whether other yeast species not belonging to *Saccharomyces* genus possess functional properties. Psomas et al. (2001) and Tiago et al. (2009) isolated from non-dairy sources (i.e., tropical fruit and plants) some strains of *D. hansenii* and *P. kluyveri* able to exhibit lipolytic and proteolytic activities, tolerate low pH and survive to simulated gastric and intestinal fluids, so as to be considered probiotics candidates. Moreover, Trotta et al. (2012) investigated the inhibition of 4-NQO by yeasts strains from wine and cheeses among which some strains of *D. hansenii*, confirming evidences about functional properties of food-borne yeasts. Acid and bile tolerance, miming gastro-intestinal transit, represent basic *in vitro* selection criteria for probiotics and a primary prerequisite of strains to exert *in situ* presumptive functional features, such as antigenotoxicity (Mishra and Prasad, 2005; Morelli, 2007). Simulated gastro-intestinal conditions carried out in this study, clearly showed that the exposure to low pH caused a relevant stress both in *L. plantarum* and yeasts strains. However, according to other studies concerning lactobacilli and yeast acid-bile tolerance (Corsetti et al., 2008; Trotta et al., 2012), a rapid viability recovery after the subsequent treatment with bile at pH 7.0 in SIF was observed. This result underlines the capability of a great number of cells to recover from viable-but-non-cultivable condition caused by SGF exposure and confirmed by high acid-bile tolerance percentage that reach in some cases 90%. The combined acid-bile tolerance observed after the *in vitro* simulation of GI conditions showed a good tolerance to endure the harsh GIT conditions, related to the ability of food-microorganisms to recover viability in the gut, as expected by selection criteria for probiotics, even if it has been reported that also dead cells can exert beneficial effects (Mottet and Michetti, 2005).

Regarding antigenotoxic activity, all the tested *L. plantarum* strains resulted active, according with Caldini et al. (2005), that found a similar high *in vitro* inhibition (>75%) of 4-NQO in strains of the same species from mozzarella cheese. In general, a similar behavior in the antigenotoxic activity was observed among the different nine yeast species. However, in three strains belonging to *D. hansenii, P. fermentans*, and *M.* aff. *fructicola* a lower antigenotoxic effect and strain dependent results were recorded, in accordance with Trotta et al. (2012) that found a similar behavior in *D. hansenii* food-borne strains.

In general, the ability to inhibit genotoxic compounds confirms that probiotic properties are features of many strains associated with foods (Holzapfel et al., 2001).

Many authors hypothesized that genotoxin deactivation was caused by interactions with microbial metabolism and structures, suggesting several possible mechanisms. However, these interactions could not be simple absorption or binding on cell wall components (Sharma and Shukla, 2016).

*Lactobacillus plantarum* and yeasts strains co-incubation with genotoxin produced evident modifications of spectroscopic properties, shifting the maximum absorbance peak of 4-NQO to lower wavelengths, as previously reported by Caldini et al. (2002, 2005) and Trotta et al. (2012). Verdenelli et al. (2010) reported that the antigenotoxic process promote the appearance of 4-amino-quinoline (an inactive compound) as a lactobacilli bioconversion product, with decrease in the content of 4-NQO. Furthermore, a recent study (Bocci et al., 2015) carried out with GC-MS and IR-Raman analyses, showed the bacterial conversion of 4-NQO in non-genotoxic conversion metabolites, mainly phenyl-quinoline and its isomers. All the above evidences confirm the hypothesis of a possible pattern of microbial conversion, that may be assumed as a bio-protective mechanism used by microorganisms to potentially prevent the DNA damage by inhibiting either inactivating the genotoxic effect of 4-NQO.

## Conclusion

The results of this study suggest for all the studied food-borne microorganisms (lactobacilli and yeasts) a good compatibility with the gut environment. In particular, many of these microorganisms show the potentiality to persist alive in the hard gut condition, and, consequently, they might have a role to express their probiotic functions. About this, antigenotoxic activity have to be considered a widespread characteristic of many food-borne *L. plantarum* and yeasts. The functional activities investigated in this paper are thought important for reducing gut pathologies and colon cancer incidence, and fermented foods commonly consumed in different types of diets could be considered to represent a vehicle for microbiota possessing potential functionality. Further studies are in progress in order to evaluate other functional and technological characteristics of the most interesting strains selected in this study for developing foods with new health-promoting properties. The information generated on food-associated potential probiotics will be helpful in improving the fermented foods and in selecting and maintaining this microbial resource.

## Author Contributions

ACo and GC designed the study. RP and ACi performed the experiments. ACo, GC, EF, RT, and RP analyzed the data, discussed the results and drafted the manuscript. All authors read and approved the final manuscript.

## Conflict of Interest Statement

The authors declare that the research was conducted in the absence of any commercial or financial relationships that could be construed as a potential conflict of interest.

## References

[B1] Ahrne S. Nobaek S. Jeppsson B. Adlerberth I. Wold A. E. Molin G. (1998). The normal *Lactobacillus* flora of healthy human rectal and oral mucosa. *J. Appl. Microbiol.* 85 88–94. 10.1046/j.1365-2672.1998.00480.x 9721659

[B2] Andrighetto C. Psomas E. Tzanetakis N. Suzzi G. Lombardi A. (2000). Randomly amplified polymorphic DNA (RAPD) PCR for the identification of yeasts isolated from dairy products. *Lett. Appl. Microbiol.* 30 5–9. 10.1046/j.1472-765x.2000.00589.x 10728551

[B3] Argyri A. A. Zoumpopoulou G. Karatzas K. A. Tsakalidou E. Nychas G. J. Panagou E. (2013). Selection of potential probiotic lactic acid bacteria from fermented olives by in vitro tests. *Food Microbiol.* 33 282–291. 10.1016/j.fm.2012.10.005 23200662

[B4] Bocci A. Sebastiani B. Trotta F. Federici E. Cenci G. (2015). *In vitro* inhibition of 4-nitroquinoline-1-oxide genotoxicity by probiotic *Lactobacillus rhamnosus* IMC501. *J. Microbiol. Biotechnol*. 25 1680–1686. 10.4014/jmb.1501.01086 26059518

[B5] Botta C. Langerholc T. Cencic A. Cocolin L. (2014). *In vitro* selection and characterization of new probiotic candidates from table olive microbiota. *PLOS ONE* 9:e94457. 10.1371/journal.pone.0094457 24714329 PMC3979845

[B6] Bourdichon F. Casaregola S. Farrokh C. Frisvad J. C. Gerds M. L. Hammes W. P. (2012). Food fermentations: microorganisms with technological beneficial use. *Int. J. Food Microbiol.* 154 87–97. 10.1016/j.ijfoodmicro.2011.12.030 22257932

[B7] Burns A. J. Rowland I. R. (2000). Anti-carcinogenicity of probiotics and prebiotics. *Curr. Issues Intest. Microbiol.* 1 13–24. 11709850

[B8] Burns A. J. Rowland I. R. (2004). Antigenotoxicity of probiotics on faecal water-induced DNA damage in human colon adenocarcinoma cells. *Mutat. Res.* 551 233–243. 10.1016/j.mrfmmm.2004.03.010 15225596

[B9] Caldini G. Trotta F. Cenci G. (2002). Inhibition of 4-nitroquinoline-1-oxide genotoxicity by *Bacillus* strains. *Res. Microbiol.* 153 165–171. 10.1016/S0923-2508(02)01302-5 12002566

[B10] Caldini G. Trotta F. Corsetti A. Cenci G. (2008). Evidence for *in vitro* anti-genotoxicity of cheese non-starter lactobacilli. *Antonie Van Leeuwenhoek* 93 51–59. 10.1007/s10482-007-9178-y 17588126

[B11] Caldini G. Trotta F. Villarini M. Moretti M. Scassellati-Sforzolini G. Cenci G. (2005). Screening of potential lactobacilli antigenotoxicity by microbial and mammalian cell-based tests. *Int. J. Food Microbiol.* 102 37–47. 10.1016/j.ijfoodmicro.2004.11.015 15925000

[B12] Capozzi V. Russo P. Dueñas M. T. López P. Spano G. (2012). Lactic Acid Bacteria producing B-group vitamins: a great potential for functional cereals products. *Appl. Microbiol. Biotechnol.* 96 1383–1394. 10.1007/s00253-012-4440-42. 23093174

[B13] Cenci G. Caldini G. Trotta F. (2005). Inhibition of DNA reactive agents by probiotic bacteria. *Appl. Microbiol. Biotechnol.* 2 102–121. 17588126

[B14] Cenci G. Caldini G. Trotta F. Bosi P. (2008). In vitro inhibitory activity of probiotic spore-forming bacilli against genotoxins. *Lett. Appl. Microbiol.* 46 331–337. 10.1111/j.1472-765X.2007.02314.x 18194161

[B15] Cenci G. Rossi J. Trotta F. Caldini G. (2002). Lactic acid bacteria isolated from dairy products inhibit genotoxic effect of 4-nitroquinoline-1-oxide in SOS chromotest. *Syst. Appl. Microbiol.* 25 483–490. 10.1078/07232020260517607 12583707

[B16] Cenci G. Trotta F. Caldini G. (2006). Tolerance to challenges miming gastrointestinal transit by spores and vegetative cells of *Bacillus clausii*. *J. Appl. Microbiol.* 101 1208–1215. 10.1111/j.1365-2672.2006.03042.x 17105550

[B17] Corsetti A. Caldini G. Mastrangelo M. Trotta F. Valmorri S. Cenci G. (2008). Raw-milk traditional Italian ewe cheeses as a source of *Lactobacillus casei* strains with acid-bile resistance and antigenotoxic properties. *Int. J. Food Microbiol.* 125 330–335. 10.1016/j.ijfoodmicro.2008.04.009 18538879

[B18] Corsetti A. Ciarrocchi A. Prete R. (2015). *Lactic Acid Bacteria: Lactobacillus* spp.: *Lactobacillus plantarum. Reference Module in Food Sciences.* Amsterdam: Elsevier 1–8.

[B19] Cunningham-Rundles S. Ahrne S. Bengmark S. Johann-Liang R. Marshall F. Metakis L. (2000). Probiotics and immune response. *Am. J. Gastroenterol.* 95(Suppl. 1) S22–S25. 10.1016/S0002-9270(99)00813-8 10634225

[B20] Czerucka D. Piche T. Rampal P. (2007). Review article: yeast as probiotics – *Saccharomyces boulardii*. *Aliment. Pharmacol. Ther.* 26 767–778. 10.1111/j.1365-2036.2007.03442.x 17767461

[B21] David L. A. Maurice C. F. Carmody R. N. Gootenberg D. B. Button J. E. Wolfe B. E (2014). Diet rapidly and reproducibly alters the human gut microbiome. *Nature* 505 559–563. 10.1038/nature12820 24336217 PMC3957428

[B22] De Los Reyes-Gàlivan C. G. Limsowtin G. K. Y. Tailliez P. Séchaud L. Accolas J. P. (1992). A *Lactobacillus helveticus* specific-DNA probe detects restriction fragment length polymorphisms in this species. *Appl. Environ. Microbiol.* 58 3429–3432. 16348794 10.1128/aem.58.10.3429-3432.1992 PMC183119

[B23] de Vries M. C. Vaughan E. E. Kleerebezem M. de Vos W. M. (2006). *Lactobacillus plantarum* – survival, functional and potential probiotic properties in the human intestinal tract. *Int. Dairy J.* 16 1018–1028. 10.1016/j.idairyj.2005.09.003

[B24] Dominici L. Villarini M. Trotta F. Federici E. Cenci G. Moretti M. (2014). Protective effects of probiotic *Lactobacillus rhamnosus* IMC501 in mice treated with PhIP. *Microbiol. Biotechnol.* 24 371–378 10.4014/jmb.1309.09072 24346468

[B25] Fann Y. C. Metosh-Dickey C. A. Winston G. W. Sygula A. Ramakrishna Rao D. N. Kadiiska M. B. (1999). Enzymatic and non-enzymatic production of free radicals from the carcinogens 4-nitroquinoline-*N*-oxide and 4-hydroxylaminoquinoline-*N*-oxide. *Chem. Res. Toxicol.* 12 450–458. 10.1021/tx980238p 10328756

[B26] Farhad M. Kailasapathy K. Tamang J. P. (2010). “Health aspects of fermented foods,” in *Fermented Foods and Beverages of the World* ed. Tamang J. P. Kailasapathy K. (New York, NY: CRC Press) 391–414. 10.1201/EBK1420094954-c15

[B27] Fleet G. H. (2007). Yeasts in foods and beverages: impact on product quality and safety. *Curr. Opin. Biotechnol.* 18 170–175. 10.1016/j.copbio.2007.01.010 17275276

[B28] Haller D. Colbus H. Gänzle M. G. Scherenbacher P. Bode C. Hammes W. P. (2001). Metabolic and functional properties of lactic acid bacteria in the gastro-intestinal ecosystem: a comparative *in vitro* study between bacteria of intestinal and fermented food origin. *Syst. Appl. Microbiol.* 24 218–226. 10.1078/0723-2020-00023 11518324

[B29] Hill C. Guarner F. Reid G. Gibson G. R. Merenstein D. J. Pot B. (2014). Expert consensus document: the international scientific association for probiotics and prebiotics consensus statement on the scope and appropriate use of the term probiotic. *Nat. Rev. Gastroenterol. Hepatol.* 11 506–514. 10.1038/nrgastro.2014.66 24912386

[B30] Holzapfel W. H. Haberer P. Snel J. Schillinger U. Huis in’t Veld J. H. (1998). Overview of gut flora and probiotics. *Int. J. Food Microbiol.* 41 85–101. 10.1016/S0168-1605(98)00044-0 9704859

[B31] Holzapfel W. H. Wood B. J. B. (2014). *Lactic Acid Bacteria: Biodiversity and Taxonomy.* New York, NY: Wiley-Blackwell 632 10.1002/9781118655252

[B32] Holzapfel W. H. Haberer P. Geisen R. Björkroth J. Schillinger U. (2001). Taxonomy and important features of probiotic microorganism in food and nutrition. *Am. J. Clin. Nutr.* 73 365S–373S. 11157343 10.1093/ajcn/73.2.365s

[B33] Jeffery I. B. O’Toole P. W. (2013). Diet-microbiota interactions and their implications for healthy living. *Nutrients* 5 234–252. 10.3390/nu5010234 23344252 PMC3571646

[B34] Kogan G. Pajtinka M. Babincova M. Miadokova E. Rauko P. Slamenova D. (2008). Yeast cell wall polysaccharides as antioxidants and antimutagens: Can they fight cancer? *Neoplasma* 55 387–393. 18665748

[B35] Kurtzman C. P. Robnett C. J. (1998). Identification and phylogeny of ascomycetous yeasts from analysis of nuclear large subunit (26S) ribosomal DNA partial sequences. *Antonie Van Leeuwenhoek* 73 331–371. 10.1023/A:1001761008817 9850420

[B36] Lisotti A. Enrico R. Mazzella G. (2013). Su2037 effects of a fermented milk containing *Kluyveromyces marxianus* B0399 and *Bifidobacterium lactis* BB12 in patients with irritable bowel syndrome: a new effective agent. *Gastroenterology* 144 538–539. 10.1016/S0016-5085(13)61999-X

[B37] Mishra V. Prasad D. N. (2005). Application on *in vitro* methods for selection of *Lactobacillus casei* strains as potential probiotics. *Int. J. Food Microbiol.* 103 109–115. 10.1016/j.ijfoodmicro.2004.10.047 16040148

[B38] Morelli L. (2007) In vitro assessment of probiotic bacteria: from survival to functionality. *Int. Dairy J.* 17 1278–1283. 10.1016/j.idairyj.2007.01.015

[B39] Moslehi-Jenabian S. Pedersen L. L. Jespersen L. (2010). Beneficial effects of probiotic and food borne yeasts on human health. *Nutrients.* 2 449–473. 10.3390/nu2040449 22254033 PMC3257658

[B40] Mottet C. Michetti P. (2005). Probiotics: wanted dead or alive. *Dig. Liver Dis.* 37 3–6. 10.1016/j.dld.2004.09.010 15702852

[B41] Nair P. Davis K. E. Shami S. Lagerholm S. (2000). The induction of SOS function in *Escherichia coli* K-12/PQ37 by 4-nitroquinoline oxide 4-NQO and fecapentaenes-12 and -14 is bile salt sensitive: implications for colon carcinogenesis. *Mutat. Res.* 447 179–185. 10.1016/S0027-5107(99)00205-5 10751601

[B42] Noomhorm A. Anal A. K. Ahmad I. (2014) “Functional foods, nutraceuticals and probiotics as functional food components” in *Functional Foods and Dietary Supplements: Processing Effects and Health Benefits* eds Noomhorm A. Anal I. Ahmad A. K. (Chichester: John Wiley & Sons, Ltd).

[B43] Novak A. Śliżwska K. Otlewska A. (2015). Antigenotoxic activity of lactic acid bacteria, prebiotics, and products of their fermentation against selected mutagens. *Regul. Toxicol. Pharmacol.* 73 938–946. 10.1016/j.yrtph.2015.09.021 26404012

[B44] Psani M. Kotzekidou P. (2006). Technological characteristics of yeast strains and their potential as starter adjuncts in Greek-style black olive fermentation. *World. J. Microbiol. Biotechnol.* 22 1329–1336. 10.1007/s11274-006-9180-y

[B45] Psomas E. Andrighetto C. Litopoulou-Tzanetaki E. Lombardi A. Tzanetakis N. (2001). Some probiotic properties of yeast isolates from infant faeces and Feta cheese. *Int. J. Food Microbiol.* 69 125–133. 10.1016/S0168-1605(01)00580-3 11589551

[B46] Quillardet P. Hofnung M. (1985). The SOS Chromotest, a colorimetric bacterial assay for genotoxins: procedures. *Mutat. Res.* 147 65–78. 10.1016/0165-1161(85)90020-2 3923333

[B47] Querol A. Barrio E. Ramon D. (1992). A comparative study of different methods of yeast strain characterization. *Syst. Appl. Microbiol.* 15 439–446. 10.1016/S0723-2020(11)80219-5

[B48] Quillardet P. Hofnung M. (1993). The SOS chromotest: a review. *Mutat. Res.* 297 235–279. 10.1016/0165-1110(93)90019-J 7692273

[B49] Qvirist L. A. De Filippo C. Strati F. Stefanini I. Sordo M. Andlid T. (2016). Isolation, identification and characterization of yeasts from fermented goat milk of the Yaghnob Valley in Tajikistan. *Front. Microbiol.* 7:1690. 10.3389/fmicb.2016.01690 27857705 PMC5093317

[B50] Raipulis J. Toma M. M. Semjonovs P. (2005). The effect of probiotics on the genotoxicity of furazolidone. *Int. J. Food Microbiol.* 102 343–347. 10.1016/j.ijfoodmicro.2004.11.029 16014301

[B51] Raman M. Ambalam P. Kondepudi K. K. Pithva S. Kothari C. Patel A. T. (2013). Potential of probiotics, prebiotics and synbiotics for management of colorectal cancer. *Gut Microbes* 4 181–192. 10.4161/gmic.23919 23511582 PMC3669163

[B52] Rosenkranz H. S. Mersch-Sundermann V. Klopman G. (1999). SOS chromotest and mutagenicity in *Salmonella*: evidence for mechanistic differences. *Mutat. Res.* 431 31–38. 10.1016/S0027-5107(99)00155-4 10656484

[B53] Ross R. P. Morgan S. Hill C. (2002). Preservation and fermentation: past, present and future. *Int. J. Food Microbiol.* 79 3–16.? 10.1016/S0168-1605(02)00174-5 12382680

[B54] Schirone M. Tofalo R. Fasoli G. Perpetuini G. Corsetti A. Manetta A. C. (2013). High content of biogenic amines in Pecorino cheeses. *Food Microbiol.* 34 137–144. 10.1016/j.fm.2012.11.022 23498190

[B55] Sharma M. Shukla G. (2016). Metabiotics: one-step ahead of probiotics; an insight into mechanisms involved in anticancerous effect in colorectal cancer. *Front. Microbiol.* 7:1940. 10.3389/fmicb.2016.01940 27994577 PMC5133260

[B56] Shenderov B. A. (2013). Metabiotics: novel idea or natural development of probiotic conception. *Microb. Ecol. Health Dis.* 24:20399. 10.3402/mehd.v24i0.20399? 23990841 PMC3747726

[B57] Silva T. Reto M. Sol M. Peito A. Peres C. M. Peres C. (2011). Characterization of yeasts from Portuguese brined olives, with a focus on their potentially probiotic behaviour. *LWT Food Sci. Technol.* 44 1349–1354. 10.1016/j.lwt.2011.01.029

[B58] Sourabh A. Kanwar S. S. Prakash Sharma O. (2011). Screening of indigenous yeast isolates obtained from traditional fermented foods of Western Himalayas for probiotic attributes. *J. Yeast Fungal Res.* 2 117–126.

[B59] Tamang J. P. Fleet G. H. (2009). “Yeasts diversity in fermented foods and beverages,” in *Yeasts Biotechnology: Diversity and Applications* eds Satyanarayana T. Kunze G. (Dordrecht: Springer) 169–198. 10.1007/978-1-4020-8292-4_9

[B60] Tamang J. P. Shin D. H. Jung S. J. Chae S. W. (2016). Functional properties of microorganisms in fermented foods. *Front. Microbiol.* 7:578 10.3389/fmicb.2016.00578 PMC4844621 27199913

[B61] Tiago F. C. P. Martins F. S. Rosa C. A. Nardi R. M. D. Cara D. C. Nicoli J. R. (2009). Physiological characterization of non-*Saccharomyces* yeasts from agro-industrial and environmental origins with possible probiotic function. *World J. Microbiol. Biotechnol.* 25 657–666. 10.1007/s11274-008-9934-9

[B62] Tofalo R. Chaves-López C. Di Fabio F. Schirone M. Felis G. E. Torriani S. (2009). Molecular identification and osmotolerant profile of wine yeasts that ferment a high sugar grape must. *Int. J. Food Microbiol.* 130 179–187. 10.1016/j.ijfoodmicro.2009.01.024 19230999

[B63] Tofalo R. Fasoli G. Schirone M. Perpetuini G. Pepe A. Corsetti A. (2014). The predominance, biodiversity and biotechnological properties of *Kluyveromyces marxianus* in the production of Pecorino di Farindola cheese. *Int. J. Food Microbiol.* 187 41–49. 10.1016/j.ijfoodmicro.2014.06.029 25038503

[B64] Torriani S. Felis G. E. Dellaglio F. (2001). Differentiation of *Lactobacillus plantarum, L. pentosus*, and *L. paraplantarum* by *recA* gene sequence analysis and multiplex PCR assay with *recA* gene-derived primers. *Appl. Environ. Microbiol.* 67 3450–3454. 10.1128/AEM.67.8.3450-3454.2001 11472918 PMC93042

[B65] Trotta F. Caldini G. Dominici L. Federici E. Tofalo R. Schirone M. (2012). Food borne yeasts as DNA-bioprotective agents against model genotoxins. *Int. J. Food Microbiol.* 153 275–280. 10.1016/j.ijfoodmicro.2011.11.009 22177230

[B66] Verdenelli M. C. Ricciutelli M. Gigli F. Cenci G. Trotta F. Caldini G. (2010). Investigation of the antigenotoxic properties of the probiotic *Lactobacillus* rhamnosus IMC 501^®^ by gas chromatography-mass spectrometry. *Ital. J. Food Sci.* 22 473–478

[B67] Walia S. Keshani S. S. Kanwar S. S. (2014). Exhibition of DNA-bioprotective activity by microflora of traditional fermented foods of North-Western Himalayas. *Food Res. Int.* 55 176–180. 10.1016/j.foodres.2013.11.001

